# Disinfection Byproducts in Drinking Water and Evaluation of Potential Health Risks of Long-Term Exposure in Nigeria

**DOI:** 10.1155/2017/7535797

**Published:** 2017-08-16

**Authors:** Nsikak U. Benson, Oyeronke A. Akintokun, Adebusayo E. Adedapo

**Affiliations:** Analytical and Environmental Chemistry Unit, Department of Chemistry, Covenant University, Km 10 Idiroko Road, Ota, Nigeria

## Abstract

Levels of trihalomethanes (THMs) in drinking water from water treatment plants (WTPs) in Nigeria were studied using a gas chromatograph (GC Agilent 7890A with autosampler Agilent 7683B) equipped with electron capture detector (ECD). The mean concentrations of the trihalomethanes ranged from zero in raw water samples to 950 *μ*g/L in treated water samples. Average concentration values of THMs in primary and secondary disinfection samples exceeded the standard maximum contaminant levels. Results for the average THMs concentrations followed the order TCM > BDCM > DBCM > TBM. EPA-developed models were adopted for the estimation of chronic daily intakes (CDI) and excess cancer incidence through ingestion pathway. Higher average intake was observed in adults (4.52 × 10^−2^ mg/kg-day), while the ingestion in children (3.99 × 10^−2^ mg/kg-day) showed comparable values. The total lifetime cancer incidence rate was relatively higher in adults than children with median values 244 and 199 times the negligible risk level.

## 1. Introduction

In sub-Saharan Africa, uncontrolled microbiological contamination of drinking water sources is a commonplace and perennially presents a significant threat to public health. Disinfection is a critical step in drinking water treatment usually performed to safeguard the public health from pathogenic microbes and waterborne diseases [1–7]. Harmful pathogens in water are destroyed by the use of disinfectants such as chlorine, chlorine dioxide, chloramine, ozone, and ultraviolet (UV) light [8–10]. However, some naturally occurring organic matter, anthropogenic contaminants, bromide, and iodide are also present in water, and when a chemical disinfectant such as chlorine is added to water, it tends to react with organic matter to form disinfection byproducts (DBPs), which are known to have adverse health effects on humans [9, 11–14]. Many disinfection byproducts (DBPs) are known for their carcinogenic, mutagenic, cytotoxic, genotoxic, or teratogenic effects [12, 13, 15–18]. However, the formation of DBPs is a function of several factors such as the pH, temperature, source water characteristics, type of disinfectant, and residence time [19–23].

The disinfection of water is an essential treatment process for safeguarding the quality of drinking water but could create undesirable chemical risk due to the formation of disinfection byproducts during chloramination, chlorination, and ozonation with natural organic matter. Since the early seventies, studies have revealed that chlorination produces potentially harmful DBPs with more than 600 DBPs detected and quantified in drinking waters [24]. Identified classes of DBPs include trihalomethanes (THMs), haloacetic acids (HAAs), haloacetaldehydes (HALs), haloketones (HKs), and nitrogenous DBPs (N-DBPs) such as haloacetonitriles (HANs), halonitromethanes (HNMs), and haloacetamides (HAcAms) [3, 25–29]. However, with the advancement in analytical methods and capabilities, emerging DBPs such as halobenzoquinones and iodotrihalomethanes have been identified [30–33]. There are two major classes of DBPs that have been regulated by US Environmental Protection Agency (USEPA) with maximum contaminant level (MCL) of 60 and 80 *μ*g/L for five HAAs and four THMs, respectively [34, 35]. The trihalomethanes are produced through the interaction of chlorine and chloramine with organic and inorganic matter in water. The THMs include bromodichloromethane (BDCM), dibromochloromethane (DBCM), bromoform (TBM), and chloroform (TCM), while the HAAs include dichloroacetic acid, trichloroacetic acid, chloroacetic acid, bromoacetic acid, and dibromoacetic acid [9, 34]. Other identified DBPs that are not regulated include the halonitromethanes, iodoacids, and other unregulated haloacids, iodotrihalomethanes, and other unregulated halomethanes, halofuranones (MX [3-chloro-4-(dichloromethyl)-5-hydroxy-2(5H)-furanone] and brominated MX DBPs), haloamides, haloacetonitriles, haloketones, tribromopyrrole, aldehydes, and N-nitrosodimethylamine (NDMA) and other nitrosamines. Possible health hazards of drinking water that contains the unregulated DBPs is however on the increase with the nonavailability of nonchlorinated or alternative disinfectants. Several epidemiological studies have reported that health complications, associated with liver, reproductive system, kidney, and central nervous system, increased risk of cancer due to consumption of drinking water that contains DBPs in excess of the maximum contaminant level (MCL) [5, 34–37].

According to documented report based on World Health Organization/UNICEF pilot study carried out in twelve states across eight hydrological areas in Nigeria, about 75% and 50% of water samples from protected dug wells and utility piped water (including water treatment plants) supplies are potentially contaminated with thermotolerant and faecal streptococci, respectively. Free chlorine was equally detected in piped water systems at levels ≥0.2 mg/l, (above maximum permissible level), which was attributed to poor dosing prior to distribution [38, 39]. In Nigeria, chlorine is widely used as the primary disinfectant because of its availability, relatively low cost, high efficiency, and convenience of application in water purification. Approximately, 99.8% of public and private drinking water treatment plants (DWTPs) in Nigeria use chlorine for disinfection [38, 40]. Although there is no recent large-scale water quality survey, which may be due to failure of government policy, negligence by responsible agency, and paucity of information regarding the occurrences of disinfection byproducts, many Nigerians may be exposed to these carcinogens through drinking water. The present study was carried out to determine the occurrence of chlorination byproducts in drinking water from four water treatment plants (WTPs) in Lagos and Ogun States, Nigeria. An attempt was also made to evaluate the potential health risks associated with possible long-term exposure to THMs through ingestion exposure pathway in adults and children.

## 2. Materials and Method

### 2.1. Sample Collection and Pretreatment

Four potable water treatment plants (WTPs) that utilize one of the two main treatment processes (chlorine-chlorine, chlorine-UV) used in Nigeria were selected for this study. Public and private WTPs in Lagos and Ogun States were selected and these include two public (OW and LW) and two private (HW and SW) water treatment plants. The codes used in the present study were adopted to protect the corporate identities of the companies involved. At the public water works, water is sourced from surface water (river) with the help of 33 kW low lift pump. Large debris and other particulate matters are prevented from entering the treatment process by coarse screens as the raw water flows into a tank. Coagulation, flocculation, and sedimentation processes are all done in a large rectangular reactor clarifier (RC). Preliming (using aluminium sulphate, Al_2_(SO_4_)_3_) and prechlorination (using 30 ppm chlorine powder) are performed simultaneously while the raw water is in the reactor clarifier. Water from the RC is pumped into several filter beds, which consist of layers of sand and gravels on top of the underdrain nozzles at the bottom of the filter beds. Any particulate matter that escapes the sedimentation process is trapped in the filter beds and thereafter directed through the underdrain nozzles designed to retain the filter media and allow the flow of water. From the filter beds, water is channeled into large reservoirs at the base of the treatment plant. Prior to the filtration process, backwashing is done to remove the debris that might have accumulated in the filter beds. This involves forcing water through the media in an opposite direction to isolate the filter from the treatment process and agitating the surface with the use of compressed air to loosen and flush the debris that has accumulated on it into the waste holding tank. A secondary disinfection involving chlorination is done to maintain a chlorine residual of 0.2 ppm as the water moves from the filter beds into reservoirs prior to distribution through an underground network of pipes for public consumption.

However, at the private water plants, raw groundwater (a borehole) is pumped into an aeration tank. It is subjected to prechlorination and preliming in the treatment tank where it is allowed to stand for six to eight hours to ensure enough contact time. Industrial filter tanks containing activated carbon and resins are employed to remove iron (Fe), odour, taste, and microsized particles from the water. The secondary disinfection is carried out by allowing the treated water to flow from the overhead tanks through the UV disinfection system, prior to packaging. The treatment goal of chlorination is to kill germs, thus inhibiting further biological activities, while improving the taste and odour of water. Raw water samples were taken directly from respective sources prior to primary disinfection process. More so, primary disinfection samples were collected from the large reservoirs after the primary disinfection stage, while the secondary water samples were obtained at the point of the distribution into pipes/packaging. Sampling of raw, primary, and secondary water samples from the public and private WTPs took place between January and May 2015. Water samples were taken in clean and well-marked 40 mL glass vials with screw caps lined with Teflon-faced septa. Each vial was filled to overflow ensuring that there are no air bubbles and headspace. After collection, 25 mg of ascorbic acid was added to each vial as a reducing agent to quench further production of disinfection byproducts (DBPs). The vials were then sealed and samples stored at 4°C prior to analyses.

### 2.2. Reagents and Solutions

All the reagents and chemicals used in this work are of HPLC grade and of highest purity. n-Pentane and n-hexane were purchased from Scharlau Chemie SA, Spain. An AccuStandard® Incorporated, USA, Commercial Stock Standard of Trihalomethanes Mix (1000 *μ*g mL^−1^) supplied with certificate of analysis was used for preparing simple and matrix-matching standard solutions for the calibration of GC-ECD instruments. Using the stock standard, nine calibration standards were prepared. Methanol and ascorbic acid used in the present work were sourced from Tedia Company Incorporated, USA. HPLC grade dichloromethane was purchased from Sigma Aldrich, USA.

### 2.3. Preparation of Internal Standard

The internal standard was prepared by dissolving 5 *µ*L dichloromethane in 10 mL hexane and well mixed by hand shaking. 50 *µ*L of this solution was added to 50 mL of pentane before the pentane was added to the sample to be extracted.

### 2.4. Extraction and Instrumental Analysis

Trihalomethanes (THMs) were isolated using a liquid-liquid extraction with HPLC grade pentane, and analyses were carried out using a gas chromatograph (GC) (7890A, Agilent, USA) with autosampler (7683B, Agilent, USA) equipped with an electron capture detector (ECD) based on USEPA method 551.1 [29]. Prior to analysis, the instrument was calibrated after preparing a multicomponent working standard from the stock standard (Stock Standard of Trihalomethanes Mix, 1000 *μ*g mL^−1^, AccuStandard Incorporated, USA) by making appropriate dilutions of the stock solution with methanol in a volumetric flask. The concentrations in ppm were chosen such that the calibration standards used between 2 and 20 *μ*L of working standard per 1000 *µ*L of methanol. In order to prepare the samples for analysis, the screw top vial was opened and 5 mL of the solution was removed. The vial was recapped and weighed to the nearest ±0.1 mg. Subsequently, 2.0 mL of pentane (with the internal standard) was added to each vial and shaken vigorously for one minute (1 min). The two phases were allowed to separate for two minutes (2 min) and a glass pipette was then used to transfer at 1 mL of the pentane (the upper phase) to a 1.8 mL screw top sample vial with a TFE septum and thereafter stored at 4°C until the sample was loaded into the autosampler for injection into the GC. The instrument was programmed to inject a 1 *μ*L aliquot of the pentane extracts into a GC equipped with a 30 m fused silica column with an internal diameter of 0.32 mm and a 1 *μ*m coating of the stationary phase DB-1 (J&W Scientific, USA). A linear flow rate of 20 cm/s is used with the following temperature program: hold for 5 min at 35°C, increase to 70°C at 10°C/min, and increase to 200°C at 20°C. Triplicate analyses were performed within 24 hours after extraction for all the samples. The calculated limit of detection (LOD) for dichlorobromomethane, dibromochloromethane, bromoform, and chloroform was ≥0.02 *µ*g/L. The recoveries of the GC-ECD method for the trihalomethanes were satisfactory and indicated analytical precisions of 99.43, 99.36, 99.48, and 99.53% for chloroform, dichlorobromomethane, dibromochloromethane, and bromoform, respectively. This ascertained the reproducibility of this method.

## 3. Results and Discussion

### 3.1. Characteristics of Raw Water Samples

The pH, temperature, chloride, total organic carbon (TOC), total dissolved solids (TDS), and residual chlorine measured for raw water samples from the private and public water treatment plants are presented in Table 1.

The raw water samples had pH values that were within the neutral pH range of 6.5 and 7.5, and the temperature ranged between 20.31 ± 0.13 and 21.88 ± 0.59°C. The chloride concentrations in the raw water samples were relatively higher in both the private and public water treatment plants. It ranged from 67.76 ± 2.89 mg/L in SW (private WTP) samples to 77.92 ± 1.57 mg/L in the public sourced raw water samples (Table 1). Chloride occurrence in enhanced concentration is regarded as a pollutant and may be introduced into surface and groundwater from anthropogenic and natural sources including industrial effluents, septic tank effluents, leachates from landfill, inorganic fertilisers from agricultural use, and saltwater intrusion into coastal inshore waters [41, 42]. The total organic carbon is essentially required as a precursor for the formation of DBPs. The TOC levels in raw water samples were not so high, ranging from 4.93 ± 0.13 to 7.34 ± 1.61 mg/L. The TDS levels, however, ranged from 48.50 ± 8.59 to 53.94 ± 5.85 mg/L. As expected, the TDS levels in raw water samples obtained from the open surface water were relatively higher than those sourced from underground water.

### 3.2. Concentrations of Trihalomethanes

The concentrations of the trihalomethanes (Table 2) in investigated water samples are presented in this section. Generally, the mean concentrations of the trihalomethanes as determined for the water samples were found to vary at different treatment stages (raw, primary, and secondary disinfection stages). The THMs were not detected in the raw water because disinfection had not been undertaken at that stage of the drinking water treatment. However, the detection of halogenated disinfection byproducts in primary and secondary water samples is likely due to the interaction of natural organic matter in raw water with chlorine disinfectant [43, 44].

The average concentrations of trihalomethanes in primary and secondary water samples from the WTPs generally followed the sequence TCM > BDCM > TBM = DBCM, which was consistent with similar documented reports [3, 5, 45, 46]. The total concentrations of the THMs (TCM + BDCM + TBM + DBCM) in primary water samples ranged from 21.92 to 997.43 *μ*g/L and 28.25 to 999.64 *μ*g/L in public and private water treatment plants, respectively. On the other hand, the TTHMs determined in water samples obtained from the secondary disinfection process ranged from 26.89 to 960.68 *μ*g/L in public WTPs and 29.20 to 950.97 *μ*g/L in private WTPs. This study shows that the total concentration of THMs found in the public and private water treatment plants during the first three months of study was relatively higher compared with published studies carried out in some countries [5, 47–50]. In general, the total concentrations of the DBPs of all primary and secondary disinfection water samples collected between January and May were above the guideline value of 0.001 mg/L stipulated by Standard Organization of Nigeria as “Nigerian Standard for Drinking Water Quality” (ICS 13.060.20) [39]. Generally speaking, there was a remarkable drop in total trihalomethanes levels in public and private water supplies between January and April, which continued into May during the sampling period. This reduction may have been attributed to the advice given to the managers of the WTPs regarding the very high concentrations of TTHMs recorded after three-months analysis, and the attempt to ensure that the levels of THMs are reduced to acceptable limit. In the present study, it was observed that TTHMs in the several treatment and distribution stages of the WTPs indicated enhanced concentrations during storage in the treatment tanks. This may be attributed to increase in TOC caused by the development of biofilms as a result of fluctuations in the water levels in the tanks.

In surface and groundwater sources, the organic matter is predominantly derived from decayed or living plant materials. This natural organic matter is present in water sources in dissolved, particulate, and colloidal forms [51]. The increased total organic carbon provides more dissolved organic matter as DBP precursor to generate more of the THMs during storage in the final treatment tanks. This is in agreement with previous reports by [50, 52].

### 3.3. Exposure Assessment

Risk assessment is a vital tool for regulation and prioritization of chemical contaminants in drinking water and could be expressed in terms of specific disease endpoints (e.g., cancer) [5, 49, 53]. However, based on the DBP distributions, an exposure assessment could be conducted to evaluate the potential intake of DBPs through multiple pathways such as inhalation and dermal and ingestion exposures. For this study, ingestion and dermal exposures were considered to be the major activity for possible contacts with THMs. In Nigeria, there is no availability of data on the shower habits of inhabitants of the investigated areas. Therefore, the estimates for shower frequency (*F*) and shower duration (*t*) were not available for computation of inhalation and dermal exposures. The chronic daily intake (CDI) estimate for the ingestion pathway was calculated by the following equation:

(1) $$\begin{array}{c} CDI_{ing}=\frac{C_{w}\times IR_{w}\times EF\times ED\times CF}{BW_{a}\times AT}, \end{array}$$

where CDI_ing_ is the value for chronic daily intake via ingestion pathway (mg/kg/day), *C*_*w*_ is the concentration of THMs in drinking water (*μ*g/L), IR_*w*_ is the water ingestion rate (L/day), EF is the exposure frequency (days/year), ED is the total exposure duration (years), AT is the averaging time (i.e., exposure duration × 365 days), and BW_*a*_ is the average body weight (kg); CF = mass conversion factor from *μ*g to mg (0.001). The reference dose, which is defined as the maximum level of the safe dose, for chloroform (TCM), bromodichloromethane (BDCM), dibromochloromethane (DBCM), and bromoform (TBM), is reported to be 0.01, 0.02, 0.02, and 0.02 mg/kg-day, respectively [54]. The average body weight for adults and children (age range 6–18 years), in Nigeria, was 70 kg and 48 kg, respectively. IR_*w*_ for adults and children were estimated at 3.3 L/day and 2.0 L/day, respectively. The exposure frequency (EF) used in the present study is estimated at 350 days/year. The ED is 52.5 years (World Bank 2013 estimate for average life expectancy of an adult in Nigeria) [55].

The chronic daily intake of THMs through ingestion route of exposure is presented in Tables 3(a) and 3(b). The highest intakes were observed in adults, while the ingestion in children aged between 6 and 18 years showed comparable values. In all water distribution systems investigated, the intakes of chloroform were highest with 4.52 × 10^−2^ and 3.99 × 10^−2^ mg/kg-day in adults and children, respectively. These high intakes may pose higher risks to human health in Nigeria.

### 3.4. Evaluation of Lifetime Cancer Incidence Rates

The lifetime incidence rates (LIR) of developing cancer from exposure to different DBPs through different pathways were calculated using (2). In addition, the total cancer incidence rate (TIR) was calculated using an additive model as shown in (3):

(2) LIRi,j=CDIi,j×SFi,j,

(3) TIR=∑i,jCDIi,j×SFi,j,

where *i* is exposure pathway, *j* is THM, and SF is slope factor. The slope factors adopted for this study were 6.10 × 10^−3^ (TCM), 6.20 × 10^−2^ (BDCM), 8.40 × 10^−2^ (DBCM), and 7.90 × 10^−2^ (TBM). Slope factors are estimated from animal toxicity data by various models, approximating 95% confidence limits. So, the calculated cancer incidence can be interpreted as the upper bound lifetime probability of an individual's developing cancer. The LIRs of developing cancer by exposure to chloroform in adults and children in Nigeria are presented in Tables 4(a) and 4(b), respectively. However, the total lifetime cancer incidence rates are presented in Table 5. Overall, the EPA mode of action (MOA) approach (a nonlinear approach) for the risk assessment on TCM set at a threshold of 0.01 mg/kg/day was not exceeded.

The median values and 5th and 95th percentiles of the cancer risk distributions from exposure to chloroform through ingestion pathway are summarized in Table 6. Results indicated that the median values of the total lifetime incidence in adults due to exposure to TCM ranged between 1.67 × 10^−4^ and 2.24 × 10^−4^ in OW and HW water disinfection systems, respectively. Also, the median values of cancer incidences in children, which could be associated to exposure to TCM, varied between 1.47 × 10^−4^ and 1.99 × 10^−4^ in OW and HW water systems. It was observed that the total lifetime incidence of developing cancer was relatively higher in adults than children. However, the highest median values in adults and children were 244 and 199 times the minimum or negligible risk level set by the USEPA (1.0 × 10^−6^), but within the regulatory limit defined by USEPA (10^−6^ to 10^−4^). Generally speaking, the presence of regulated DBPs in drinking water highlights the problems associated with chlorination treatment process, which can potentially increase the human exposure to these cancer-causing contaminants. Again, these results support the premise that carcinogenic organic compounds and toxic metals in drinking water and groundwater sources in the country are largely found at concentrations above threshold limits [38, 40, 56].

## 4. Conclusion and Recommendations

In this study, the concentration of trihalomethanes was assessed in drinking water from four different water treatment plants (HW, OW, LW, and SW) in two heavily populated states in Nigeria. The levels of the total trihalomethanes were found to be higher at the initial stage of the research but gradually reduced probably due to more careful handling of the water treatment processes as the quality control officers were briefed on the results of previous analysis when the next samples were being collected. This is an indication that the treatment processes handling contributes a great deal to the formation of disinfection by-products in drinking water. This has to do partly with carefulness in the addition of chlorine, which is mostly used in all the water treatment plants in the sampled areas; the documented dosage is 20–30 mg/L but it does not seem this is strictly adhered to. In addition to this are the processes of sedimentation/flocculation and the maintenance of the right amount of residual chlorine in the distribution system.

For the water treatment plants to achieve the SON (0.001 mg/L) and USEPA (0.08 mg/L) recommended maximum permissible levels (MCL) for the TTHMs, a constant effort is required to reduce the concentrations of all the DBPs to the barest minimum. This can be achieved by protecting the source water from excessive pollution thereby reducing the DBP precursors. The use of chloramine as disinfectant has proven to produce very minimal amount of DBPs in countries like Europe, America, and Australia. Therefore, a switch from chlorine usage during primary disinfection by DWTPs in Nigeria to chloramine is strongly recommended with ultraviolet light as the secondary disinfectant. Operators of public and private water treatment plants should adopt viable alternative primary disinfection strategies such as using ClO_2_, ozone, and UV disinfection to chlorination. In addition, a thorough evaluation of the efficiency of WTPs and operational practices of the water treatment processes and the piped distribution network is proposed to examine the possible reasons for the exceedances of TTHMs.

## Figures and Tables

**Table 1:** Summary statistics for raw water samples collected from private and public water treatment plants.

	pH	Temp. (°C)	Chloride (mg/L)	TDS (mg/L)	TOC (mg/L)	Res. Cl (mg/L)
HWR	6.78 ± 0.16	20.31 ± 0.13	70.09 ± 0.26	48.50 ± 8.59	4.93 ± 0.13	0.00
SWR	6.91 ± 0.14	20.79 ± 0.06	67.76 ± 2.89	49.70 ± 8.86	5.12 ± 0.89	0.00
OWR	6.90 ± 0.15	21.88 ± 0.59	77.92 ± 1.57	52.32 ± 5.33	6.52 ± 1.50	0.00
LWR	6.98 ± 0.13	21.72 ± 0.52	75.88 ± 1.78	53.94 ± 5.85	7.34 ± 1.61	0.00

**Table 2:** Average concentration (*μ*g/L) (*n* = 3) of trihalomethanes in raw, primary, and secondary water samples from WTPs obtained between January and May 2015.

Source	TCM	BDCM	DBCM	TBM	TTHMs
Jan.					
OWR	BD	BD	BD	BD	0
OWP	997.43	BD	BD	0.40	997.83
OWS	960.68	BD	BD	BD	960.68
LWR	BD	BD	BD	BD	0
LWP	716.04	0.42	0.38	BD	716.84
LWS	825.04	BD	BD	BD	825.04
HWR	BD	BD	BD	BD	0
HWP	755.70	BD	BD	BD	755.70
HWS	812.35	BD	BD	BD	812.35
SWR	BD	BD	BD	BD	0
SWP	999.64	BD	BD	BD	999.64
SWS	950.97	BD	BD	BD	950.97

Feb.					
OWR	BD	BD	BD	BD	0
OWP	953.77	BD	BD	BD	953.77
OWS	887.34	BD	BD	BD	887.34
LWR	BD	BD	BD	BD	0
LWP	900.70	0.42	BD	BD	901.12
LWS	825.04	BD	BD	BD	825.04
HWR	BD	BD	BD	BD	0
HWP	916.94	BD	BD	BD	916.94
HWS	872.00	BD	BD	BD	872.00
SWR	BD	BD	BD	BD	0
SWP	906.38	BD	BD	BD	906.38
SWS	712.50	BD	BD	BD	712.50

Mar.					
OWR	BD	BD	BD	BD	0
OWP	561.86	BD	BD	BD	561.86
OWS	582.32	0.41	BD	BD	582.73
LWR	BD	BD	BD	BD	0
LWP	620.55	BD	BD	BD	620.55
LWS	846.79	0.41	BD	BD	847.20
HWR	BD	BD	BD	BD	0
HWP	777.70	BD	BD	BD	777.70
HWS	803.42	BD	BD	BD	803.42
SWR	BD	BD	BD	BD	0
SWP	624.54	BD	BD	BD	624.54
SWS	591.99	BD	BD	BD	591.99

Apr.					
OWR	BD	BD	BD	BD	0
OWP	31.21	BD	BD	BD	31.21
OWS	28.34	0.41	BD	BD	28.75
LWR	BD	BD	BD	BD	0
LWP	21.92	BD	BD	BD	21.92
LWS	28.44	BD	BD	BD	28.44
HWR	BD	BD	BD	BD	0
HWP	28.25	BD	BD	BD	28.25
HWS	30.03	BD	BD	BD	30.03
SWR	BD	BD	BD	BD	0
SWP	28.87	BD	BD	BD	28.87
SWS	32.11	BD	BD	BD	32.11

May					
OWR	BD	BD	BD	BD	0
OWP	30.45	BD	BD	BD	30.45
OWS	34.15	BD	BD	BD	34.15
LWR	BD	BD	BD	BD	0
LWP	27.20	BD	BD	BD	27.20
LWS	26.89	BD	BD	BD	26.89
HWR	BD	BD	BD	BD	0
HWP	32.90	BD	BD	BD	32.90
HWS	29.20	BD	BD	BD	29.20
SWR	BD	BD	BD	BD	0
SWP	32.50	BD	BD	BD	32.50
SWS	32.10	BD	BD	BD	32.10

R: raw, P: primary, S: secondary disinfection samples, and BD: below detection limit.

**Table:** (a) Exposure assessment of THMs (mg/kg-day) through ingestion in adults

Sites	Jan.			Feb.			March			April			May		
	TCM × 10^−2^	BDCM × 10^−5^	DBCM × 10^−5^	TCM × 10^−2^	BDCM × 10^−5^	DBCM	TCM × 10^−2^	BDCM × 10^−5^	DBCM	TCM × 10^−3^	BDCM × 10^−5^	DBCM	TCM × 10^−3^	BDCM	DBCM
OWP	4.51	—	—	4.31	—	—	2.54	—	—	1.41	—	—	1.37	—	—
OWS	4.34	—	—	4.01	—	—	2.63	1.85	—	1.28	1.85	—	1.54	—	—
LWP	3.23	1.89	1.72	4.07	1.89	—	2.81	—	—	9.90	—	—	1.23	—	—
LWS	3.73	—	—	3.72	—	—	3.83	—	—	1.29	—	—	1.21	—	—
HWP	3.41	—	—	4.15	—	—	3.52	1.85	—	1.27	—	—	1.49	—	—
HWS	3.67	—	—	3.94	—	—	3.63	—	—	1.36	—	—	1.32	—	—
SWP	4.52	—	—	4.09	—	—	2.63	—	—	1.30	—	—	1.47	—	—
SWS	4.29	—	—	3.22	—	—	2.67	—	—	1.45	—	—	1.45	—	—

**Table:** (b) Exposure assessment of THMs (mg/kg-day) through ingestion in children (6–18 years)

Sampling sites	Jan.			Feb.			March			April			May		
	TCM × 10^−2^	BDCM × 10^−5^	DBCM × 10^−5^	TCM × 10^−2^	BDCM × 10^−5^	DBCM	TCM × 10^−2^	BDCM × 10^−5^	DBCM	TCM × 10^−3^	BDCM × 10^−5^	DBCM	TCM × 10^−3^	BDCM	DBCM
OWP	3.98	—	—	3.81	—	—	2.24	—	—	1.25	—	—	1.22	—	—
OWS	3.83	—	—	3.55	—	—	2.32	1.64	—	1.13	1.64	—	1.36	—	—
LWP	2.86	1.68	1.51	3.59	1.67	—	2.48	—	—	0.87	—	—	1.09	—	—
LWS	3.29	—	—	3.29	—	—	3.38	—	—	1.13	—	—	1.07	—	—
HWP	3.01	—	—	3.66	—	—	3.10	1.64	—	1.12	—	—	1.31	—	—
HWS	3.25	—	—	3.48	—	—	3.21	—	—	1.19	—	—	1.17	—	—
SWP	3.99	—	—	3.62	—	—	2.49	—	—	1.15	—	—	1.29	—	—
SWS	3.79	—	—	2.84	—	—	2.36	—	—	1.28	—	—	1.28	—	—

**Table:** (a) Lifetime incidence rates of developing cancer by exposure to chloroform in adults

Sampling sites	Jan.	Feb.	Mar.	Apr.	May
OWP	2.7504*E* − 04	2.6301*E* − 04	1.6043*E* − 06	8.6062*E* − 06	8.3966*E* − 06
OWS	2.6491*E* − 04	2.4468*E* − 04	1.4925*E* − 06	7.8148*E* − 06	9.4169*E* − 06
LWP	1.9745*E* − 04	2.4837*E* − 04	1.5150*E* − 06	6.0445*E* − 06	7.5004*E* − 06
LWS	2.2750*E* − 04	2.2750*E* − 04	1.3878*E* − 06	7.8424*E* − 06	7.4150*E* − 06
HWP	2.0838*E* − 04	2.5285*E* − 04	1.5423*E* − 06	7.7900*E* − 06	9.0722*E* − 06
HWS	2.2401*E* − 04	2.4045*E* − 04	1.4667*E* − 06	8.2808*E* − 06	8.0520*E* − 06
SWP	2.7565*E* − 04	2.4993*E* − 04	1.5246*E* − 06	7.9610*E* − 06	8.9619*E* − 06
SWS	2.6223*E* − 04	1.9647*E* − 04	1.1984*E* − 06	8.8544*E* − 06	8.8516*E* − 06

**Table:** (b) Lifetime incidence rates of developing cancer by exposure to chloroform in children

Sampling sites	Jan.	Feb.	Mar.	Apr.	May
OWP	2.4309*E* − 04	2.3245*E* − 04	1.3693*E* − 04	7.6065*E* − 06	7.4213*E* − 06
OWS	2.3414*E* − 04	2.1626*E* − 04	1.4192*E* − 04	6.9070*E* − 06	8.3230*E* − 06
LWP	1.7451*E* − 04	2.1952*E* − 04	1.5124*E* − 04	5.3424*E* − 06	6.6292*E* − 06
LWS	2.0108*E* − 04	2.0108*E* − 04	2.0638*E* − 04	6.9314*E* − 06	6.5536*E* − 06
HWP	1.8418*E* − 04	2.2347*E* − 04	1.8954*E* − 04	6.8851*E* − 06	8.0184*E* − 06
HWS	1.9798*E* − 04	2.1252*E* − 04	1.9581*E* − 04	7.3189*E* − 06	7.1166*E* − 06
SWP	2.4363*E* − 04	2.2090*E* − 04	1.5221*E* − 04	7.0362*E* − 06	7.9209*E* − 06
SWS	2.3177*E* − 04	1.7365*E* − 04	1.4428*E* − 04	7.8259*E* − 06	7.8234*E* − 06

**Table 5:** Total cancer incidence rate (×10^−4^) by exposure to chloroform in adults and children.

	Jan.		Feb.		Mar.		Apr.		May	
	ADT	CHD	ADT	CHD	ADT	CHD	ADT	CHD	ADT	CHD
OW	5.3996	4.7723	5.0769	4.4872	3.1551	2.7886	0.1642	0.1451	0.1781	0.1574
LW	4.2496	3.7559	4.7588	4.2060	4.0662	3.5762	0.1386	0.1227	0.1491	0.1318
HW	4.3240	3.8217	4.9331	4.3600	4.3600	3.8535	0.1607	0.1420	0.1420	0.1513
SW	5.3789	4.7541	4.4641	3.9456	3.3546	2.9649	0.1682	0.1486	0.1486	0.1574

**Table 6:** Excess cancer incidences from exposure to TCM through different ingestion (×10^−4^) in adults and children.

	OW	LW	HW	SW
Adults	1.67 (0.17, 5.32)	2.11 (0.14, 4.63)	2.24 (0.15, 4.79)	1.76 (0.15, 5.15)
Children	1.47 (0.15, 4.700)	1.85 (0.13, 4.09)	1.99 (0.14, 4.23)	1.56 (0.15, 4.55)

Data shown are median values of the risk distributions, and values in the parentheses are the 5th and 95th percentiles of the risk distributions.
